# EDITORIAL COMMENT: ARTIFICIAL URINARY SPHINCTER REVISION FOR URETHRAL ATROPHY: COMPARING SINGLE CUFF DOWNSIZING AND TANDEM CUFF PLACEMENT

**DOI:** 10.1590/S1677-5538.IBJU.2016.0240.1

**Published:** 2017

**Authors:** Márcio Augusto Averbeck

**Affiliations:** 1 Hospital Moinhos de Vento Hospital, Porto Alegre, Brasil

Several therapeutic options have been proposed for patients with recurrent or persistent post-prostatectomy urinary incontinence due to urethral atrophy: changing the balloon reservoir for a higher pressure one, downsizing the cuff diameter, or increasing the amount of fluid in the system (1-3). Theoretically, a transcorporal cuff could possibly provide some supplementary bulk of tissue to the circumference of the urethra, possibly decreasing the risk of erosion (4).

This article Linder, et al. (5) deals with a matter of great clinical interest. This is a retrospective series reporting 69 cases of revision surgeries for urethral atrophy, of which 56 (82%) underwent tandem cuff placements, 12 (18%) underwent single cuff downsizings and one case had a single cuff relocated proximally. There was no difference in 3-year overall device survival compared between single cuff and tandem cuff revisions (60% vs. 76%, p=0.94). Of the 56 tandem cuff placements, 8 (14%) were performed with transcorporal approach. Interestingly, these patients had adverse 3-year device survival compared to those without a transcorporal approach (44% vs. 80%, p=0.0016). Despite of the inherent limitations of this retrospective study, it seems that a transcorporal approach should be reserved for very selected patients (most probably in secondary or tertiary interventions). Randomized controlled trials are still needed to guide what is the best technique for each group of patients, taking into account anatomical characteristics, previous radiation therapy, the risk of urethral erosion and other local complications.

## References

[1] Barrett DM, Licht MR, Walsh PC, Retik AB, Vaughan ED, Wein AJ (1998). Implantation of the artificial genitourinary sphincter in men and women. Campbell’s Urology.

[2] Kabalin JN (1996). Addition of a second urethral cuff to enhance performance of the artificial urinary sphincter. J Urol.

[3] Petrou SP, Williams HJ JR, Young PR (2001). Radiographic imaging of the artificial urinary sphincter pressure regulating balloon. J Urol.

[4] Guralnick ML, Miller E, Toh KL, Webster GD (2002). Transcorporal artificial urinary sphincter cuff placement in cases requiring revision for erosion and urethral atrophy. J Urol.

[5] Linder BJ, Viers BR, Ziegelmann MJ, Rivera ME, Elliott DS (2016). Artificial urinary sphincter revision for urethral atrophy: Comparing single cuff downsizing and tandem cuff placement. Int Braz J Urol.

